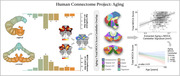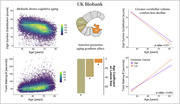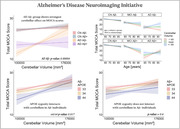# The Cerebellum Plays a Protective Role in Cognitive Aging and Disease: Insights from a Multi‐Cohort Study

**DOI:** 10.1002/alz.085743

**Published:** 2025-01-09

**Authors:** Federico d’Oleire Uquillas, Esra Sefik, Jakob Seidlitz, Jewel Merriman, Veronica Zhang, Mikhail Kislin, Jonathan D Cohen, Aaron Alexander‐Bloch, Richard A.I. Bethlehem, Jorge Sepulcre, Samuel S.‐H. Wang, Patrizia Vannini, Jesse Gomez, the Alzheimer's Disease Neuroimaging Initiative

**Affiliations:** ^1^ Princeton University, Princeton, NJ USA; ^2^ Department of Child and Adolescent Psychiatry and Behavioral Science, The Children’s Hospital of Philadelphia, Philadelphia, PA USA; ^3^ Penn/CHOP Lifespan Brain Institute, University of Pennsylvania, Philadelphia, PA USA; ^4^ University of Cambridge, Cambridge UK; ^5^ Massachusetts General Hospital, Boston, MA USA; ^6^ Gordon Center for Medical Imaging, Massachusetts General Hospital, Boston, MA USA; ^7^ Athinoula A. Martinos Center for Biomedical Imaging, Charlestown, MA USA; ^8^ Brigham and Women's Hospital, Harvard Medical School, Boston, MA USA; ^9^ Massachusetts General Hospital, Harvard Medical School, Boston, MA USA

## Abstract

**Background:**

Studying brain reserve — the brain’s resilience to age‐related changes or damage — is crucial for understanding protective mechanisms against cognitive decline. The cerebellum may be a key region in brain reserve, but it has been historically understudied. This investigation delves into this critical area within the largest aging multi‐cohort to date.

**Methods:**

Data were sourced from the Human Connectome Project‐Aging (n=708, 36‐100yrs), UK Biobank (n=45,013, 44‐81yrs), and ADNI (n=1,441, 56‐95yrs). ADNI participants were cognitively normal, had mild cognitive impairment, or Alzheimer’s disease (AD) dementia. We examined associations between cerebellar tissue volume, age, Montreal Cognitive Assessment (MOCA) scores, global PET amyloid burden, and APOE genotype. Statistical models included sex and estimated intracranial volume (eTIV) as covariates, with adjusted household income considered when available.

**Results:**

Spatial analysis of HCP‐Aging data revealed that aging unfolds heterogeneously across the cerebellum; posterior portions of the cerebellum (Crus I) showed greater effects than anterior regions (Lobule I‐III) (Bonferroni‐corrected, p<0.05). MOCA scores were associated with higher tissue density in the cerebellum (p<0.0001), as much as neocortex, and MOCA scores coupled most strongly with posterior cerebellar cortex, overlapping frontoparietal, ventral attention, and default mode network representations. Strikingly, greater volume in this MOCA‐related cerebellar cortex signature protected against aging‐related cognitive decline (p=0.029). We validate tissue aging results in the UK Biobank – again, posterior cerebellum demonstrated the greatest aging effect (p<0.0001), and individuals with greater cerebellar volumes showed less decline in processing speed and executive function (Trails Making‐B time: p=0.001; Digit Symbol Substitution score: p=0.038). Across all ADNI diagnostic groups, AD patients with low amyloid‐beta burden (Aβ‐) exhibited the strongest cerebellar association with MOCA (volume x group, Aβ‐ AD: p=0.00004). In Aβ‐ individuals, APOE genotype interacted with cerebellar volume on MOCA, with ε4/ε4 carriers showing the greatest effect (volume x APOE, ε4/ε4: p=0.017).

**Conclusions:**

Our large‐scale study demonstrates the cerebellum’s critical role in mitigating patterns of cognitive decline. This protection persists until significant amyloid burden, especially in APOE ε4/ε4 carriers, reshaping our understanding of reserve and AD risk. The cerebellum’s integral contribution to brain reserve has substantial implications for aging populations and should be a focus of future clinical research.